# Adaptive Response and Tolerance to Acetic Acid in *Saccharomyces cerevisiae* and *Zygosaccharomyces bailii*: A Physiological Genomics Perspective

**DOI:** 10.3389/fmicb.2018.00274

**Published:** 2018-02-21

**Authors:** Margarida Palma, Joana F. Guerreiro, Isabel Sá-Correia

**Affiliations:** Institute for Bioengineering and Biosciences, Department of Bioengineering, Instituto Superior Técnico, Universidade de Lisboa, Lisbon, Portugal

**Keywords:** *Saccharomyces cerevisiae*, *Zygosaccharomyces bailii*, weak acid food preservatives, acetic acid adaptive response, acetic acid tolerance, physiological genomics

## Abstract

Acetic acid is an important microbial growth inhibitor in the food industry; it is used as a preservative in foods and beverages and is produced during normal yeast metabolism in biotechnological processes. Acetic acid is also a major inhibitory compound present in lignocellulosic hydrolysates affecting the use of this promising carbon source for sustainable bioprocesses. Although the molecular mechanisms underlying *Saccharomyces cerevisiae* response and adaptation to acetic acid have been studied for years, only recently they have been examined in more detail in *Zygosaccharomyces bailii*. However, due to its remarkable tolerance to acetic acid and other weak acids this yeast species is a major threat in the spoilage of acidic foods and beverages and considered as an interesting alternative cell factory in Biotechnology. This review paper emphasizes genome-wide strategies that are providing global insights into the molecular targets, signaling pathways and mechanisms behind *S. cerevisiae* and *Z. bailii* tolerance to acetic acid, and extends this information to other weak acids whenever relevant. Such comprehensive perspective and the knowledge gathered in these two yeast species allowed the identification of candidate molecular targets, either for the design of effective strategies to overcome yeast spoilage in acidic foods and beverages, or for the rational genome engineering to construct more robust industrial strains. Examples of successful applications are provided.

## Introduction

The yeast *Saccharomyces cerevisiae* plays an essential role in the production of foods (e.g., bread) and alcoholic beverages (e.g., wine and beer). However, this yeast species is also a food spoilage agent, being able to overcome several harsh conditions that are employed in the food industry to maintain the microbial stability of its products and avoid undesirable changes in their organoleptic properties (James and Stratford, 2003).

Yeasts belonging to the genus *Zygosaccharomyces* are associated with a detrimental role in food and beverage industries, being considered the most problematic food spoilage yeasts. In fact, they are able to adapt and proliferate in the presence of extremely high concentrations of weak acids (*Zygosaccharomyces bailii* and *Zygosaccharomyces lentus*), sugar and salt (*Z. rouxii*) compared to those tolerated by other spoilage yeasts (James and Stratford, 2003). Within the genus, *Z. bailii* stands out as the most problematic spoilage yeast, mainly in acidified food products, such as mayonnaise, salad dressings, fruit concentrates and various non-carbonated fruit drinks, also being frequently isolated in wine due to its tolerance to both organic acids at low pH and ethanol (Thomas and Davenport, 1985; James and Stratford, 2003). *Z. bailii* is also an emerging spoiler of new food types such as mustards and fruit-flavored carbonated soft drinks (Sá-Correia et al., 2014). The remarkable tolerance of *Z. bailii* to weak acid food preservatives allows growth to occur in food products with concentrations above those legally permitted (Sá-Correia et al., 2014). Depending on the food product, the limit concentrations approved for use of sorbic and benzoic acids as food additives mainly range from 0.5 to 2 g/L (European Commission, 2011). Concerning the use of acetic acid as a food additive, the concentration is *quantum satis* (European Commission, 2011) this meaning that acetic acid should be used in food products under conditions that do not result in consumer’s deception. In the case of *Z. bailii*, the average minimum inhibitory concentration (MIC) determined for several strains is approximately 8 and 10 g/L (pH 4.0) for sorbic and benzoic acids, respectively, and around 28 g/L (pH 4.0) for acetic acid, which are much higher than the values commonly determined for *S. cerevisiae* (Stratford et al., 2013b). Those different tolerance levels are highly relevant also because, despite their widespread use and classification as “generally recognized as safe” (GRAS), weak acids may cause intolerance (Joneja, 2003; Stratford, 2006; Theron and Lues, 2010).

Acetic acid is also an important inhibitory byproduct of alcoholic fermentation carried out by *S. cerevisiae* (Garay-Arroyo et al., 2004; Graves et al., 2006) and can achieve levels that, combined with high concentrations of ethanol and other toxic metabolites, may lead to fermentation arrest or reduced ethanol productivity (Rasmussen et al., 1995; Garay-Arroyo et al., 2004; Graves et al., 2006). Moreover, acetic acid is a highly important inhibitory compound in the context of lignocellulosic hydrolysates-based bioethanol production where its presence may seriously affect fermentation performance (Jönsson et al., 2013). Concentrations of acetic acid in lignocellulosic hydrolysates strongly depend on the feedstock and on the severity of the pretreatment (Jönsson et al., 2013). Levels of 3.4 g/L (pH 5.0) can, for instance, be achieved in wheat straw hydrolysates (Olofsson et al., 2010). Although these concentrations are below *S. cerevisiae* MIC for acetic acid (around 9 g/L at pH 4.0) (Stratford et al., 2013b), it is the combined effect of acetic acid and several other compounds produced during pretreatment of lignocellulosic hydrolysates that inhibits *S. cerevisiae* fermentation performance. It is therefore essential to understand the mechanisms underlying *S. cerevisiae* tolerance to acetic acid in order to develop robust industrial strains.

Considering the importance of acetic acid as a yeast growth inhibitor in modern Biotechnology and Food Industry, this review paper provides an updated critical review of scientific literature on the adaptive response and tolerance to this weak acid emphasizing the physiological toxicogenomics perspective. The understanding of yeast physiology exploring functional and comparative genomic strategies allows a holistic assessment of the complex adaptive responses to environmental stresses and the identification of tolerance or susceptibility determinants to these stresses at a genome-wide scale. Yeast physiological toxicogenomics is thus instrumental to guide synthetic pathway engineering and other approaches for cell robustness manipulation, either for the sustainable production of fuels and chemicals or for the control of spoiling yeasts.

## Mechanisms Underlying the Adaptive Response and Tolerance to Acetic Acid in Yeasts

### The Physiological Genomic Approaches

Upon exposure to inhibitory, but sublethal, concentrations of acetic acid, yeast cells may enter a more or less extended period of growth arrest, but after this adaptation period exponential growth is resumed with a lower maximum specific growth rate (Fernandes et al., 2005; Guerreiro et al., 2012). On the other hand, lethal concentrations of acetic acid may induce regulated cell death (RCD), either by apoptosis or necrosis, depending on the severity of acetic acid stress (Ludovico et al., 2001, 2003).

**FIGURE 1:**
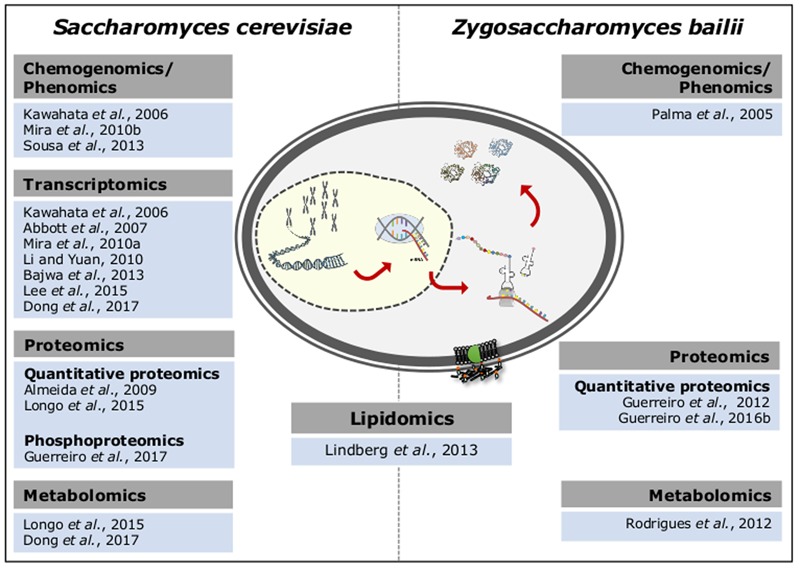
Functional genomics-based approaches explored to obtain mechanistic insights into the adaptive response and tolerance to sublethal and lethal concentrations of acetic acid in *S. cerevisiae* and *Z. bailii.*

After two decades of post-genomic research in *S. cerevisiae*, a more comprehensive understanding of the molecular mechanisms underlying this species response and adaptation to sublethal or lethal concentrations of acetic acid was obtained through the integration of several functional genomic approaches (**Figure 1**). When compared to *S. cerevisiae*, the number of Omic-based approaches applied to *Z. bailii* is still limited (**Figure 1**). Among other reasons, the lack of a *Z. bailii* genome sequence with suitable annotation has limited those studies for years. However, since 2013 the annotated genome sequences of two *Z. bailii* strains (Galeote et al., 2013; Palma et al., 2017b) and two hybrid strains resulting from *Z. bailii* and an unidentified *Zygosaccharomyces* species were also disclosed (Mira et al., 2014; Ortiz-Merino et al., 2017). This genome data has largely accelerated the understanding of *Z. bailii* species as a biological system with interesting genetic and physiological traits. Moreover, it has provided genomic information essential to study the mechanisms underlying *Z. bailii* tolerance to acetic acid, yet to be explored.

In *S. cerevisiae*, two chemogenomics screenings of commercial haploid yeast mutant collections containing thousands of single deletion mutants in non-essential yeast genes were useful to identify candidate molecular determinants and mechanisms of tolerance to sublethal concentrations of acetic acid (Kawahata et al., 2006; Mira et al., 2010b). These screenings, that were performed under different experimental conditions, led to the identification of a number of genes required for maximum tolerance to acetic acid, but the two datasets only share a total of 150 genes (Mira et al., 2010c). These include, for example, genes involved in intracellular pH homeostasis, vacuolar transport, positive regulation of cellular processes, amino acid and carbohydrate metabolism (Kawahata et al., 2006; Mira et al., 2010b). Metabolic pathways that regulate RCD in *S. cerevisiae* response to lethal concentrations of acetic acid were also revealed based on a chemogenomics study, with carbohydrate metabolism emerging as an essential regulator of RCD (Sousa et al., 2013).

Given the unavailability of an equivalent single deletion mutant collection for *Z. bailii*, the identification of the determinants of tolerance to acetic acid was attempted using a genomic library of the *Z. bailii*-derived hybrid strain ISA1307 (Palma et al., 2015). This library was used to rescue the high acetic acid susceptibility phenotype of *S. cerevisiae haa1* deletion mutant (Palma et al., 2015). *Z. bailii* genes putatively involved in cellular transport and transport routes, protein fate, protein synthesis, amino acid metabolism and transcription were proposed as strong candidate determinants of acetic acid tolerance in *Z. bailii* (Palma et al., 2015).

The transcriptional alterations occurring in *S. cerevisiae* cells challenged with acetic acid stress were examined in several studies performed under different experimental settings, such as different cell growth phase, acetic acid concentration and medium composition and pH (Kawahata et al., 2006; Abbott et al., 2007; Li and Yuan, 2010; Mira et al., 2010a; Bajwa et al., 2013; Lee et al., 2015; Dong et al., 2017). Transcriptional profiling of the early response of *S. cerevisiae* cells to acetic acid (Kawahata et al., 2006; Li and Yuan, 2010; Mira et al., 2010a) or after adaptation to this weak acid (Kawahata et al., 2006; Bajwa et al., 2013; Lee et al., 2015) were described. Response to acetic acid of steady-state anaerobic and glucose-limited chemostat *S. cerevisiae* cultures was also investigated (Abbott et al., 2007). Although the genes identified in different studies as up-regulated under acetic acid stress do not fully overlap, a few genes coding for plasma membrane proteins or proteins of a still unidentified function emerged in, at least, three of those works.

A quantitative proteomics analysis based on two-dimensional gel electrophoresis (2-DE) contributed to the identification of the alterations in the protein content of *Z. bailii* hybrid strain ISA1307 occurring in response to sudden exposure or during exponential growth in the presence of an inhibitory sublethal concentration of acetic acid (Guerreiro et al., 2012). The increased content of a particular set of proteins suggested that, in the presence of glucose, acetate is channeled into the tricarboxylic acid cycle, being co-consumed with glucose. These results were corroborated by a metabolomics study where the biochemical pathways associated with acetic acid utilization during co-metabolism with glucose were investigated in the same strain (Rodrigues et al., 2012). In *Z. bailii*, a quantitative proteomics study for the analysis of expression of mitochondrial proteins in cells exposed to lethal concentrations of acetic acid highlighted the importance of metabolic and energy processes, in particular of mitochondrial energetic metabolism, in acetic acid-induced RCD response (Guerreiro et al., 2016b). Cellular processes like oxidative stress response, protein translation, amino acid (in particular glutamate) and nucleotide metabolism, among others, were also found to be involved in this cellular response (Guerreiro et al., 2016b). In *S. cerevisiae*, quantitative proteomics was used to examine yeast response to a lethal concentration of acetic acid, revealing alterations in the levels of proteins implicated in the general amino-acid control system, further shown to be associated with a severe intracellular amino-acid starvation, as well as in the Target-of-rapamycin (TOR) pathway (Almeida et al., 2009). Moreover, quantitative proteomic and metabolomic analyses of *S. cerevisiae* parental and derived deletion mutant *yca1* strains in acetic acid-induced RCD identified significant alterations in carbohydrate catabolism, lipid metabolism, proteolysis and stress-response, thus emphasizing the importance of Yca1 metacaspase in RCD caused by lethal concentrations of acetic acid (Longo et al., 2015).

The alterations occurring at the level of the membrane ph1osphoproteome during yeast early adaptive response to a sublethal concentration of acetic acid stress and the role played by the Hrk1 kinase in such response have recently been investigated (Guerreiro et al., 2017). Hrk1 is a protein kinase belonging to the “Npr1-family” of kinases dedicated to the regulation of plasma membrane transporters that was identified in previous Omics approaches as a determinant of acetic acid tolerance and involved in yeast response to acetic acid stress (Abbott et al., 2007; Mira et al., 2010a,b). The investigation of membrane phosphoproteome hinted toward the contribution of phosphorylation in the regulation of processes related with translation, protein folding and processing, transport, and cellular homeostasis in yeast response to acetic acid stress (Guerreiro et al., 2017).

The studies previously mentioned were exclusively dedicated to either *S. cerevisiae* or Z. *bailii* under acetic acid stress. In fact, high-throughput comparisons between *S. cerevisiae* and *Z. bailii* using similar experimental conditions are scarce in the context of acetic acid stress. One relevant exception is the lipidomic profiling of the major lipid species found in the plasma membrane of exponentially growing cells of both species under basal and acetic acid stress conditions (Lindberg et al., 2013). The correlation between the higher basal level of complex sphingolipids in *Z. bailii* when compared to *S. cerevisiae*, and consequent reduced plasma membrane permeability to acetic acid, was one of the most important findings in this study (Lindberg et al., 2013).

The exploitation of functional genomics approaches and tools, besides providing an integrative view on how yeast cells respond to a challenging environment, has strongly contributed to the understanding of the molecular players involved in such response. Nevertheless, the combined use of such global approaches with more focused molecular and cellular biology studies is essential to understand in depth the complexity of the mechanisms underlying the response and tolerance to a particular stress. In the following sections, the main molecular mechanisms underlying *S. cerevisiae* and *Z. bailii* response and tolerance to sublethal and lethal concentrations of acetic acid are reviewed based on genome-wide and high-throughput analyses complemented by more detailed molecular, biochemical and physiological studies.

### Acetic Acid Uptake and Toxicity

**FIGURE 2:**
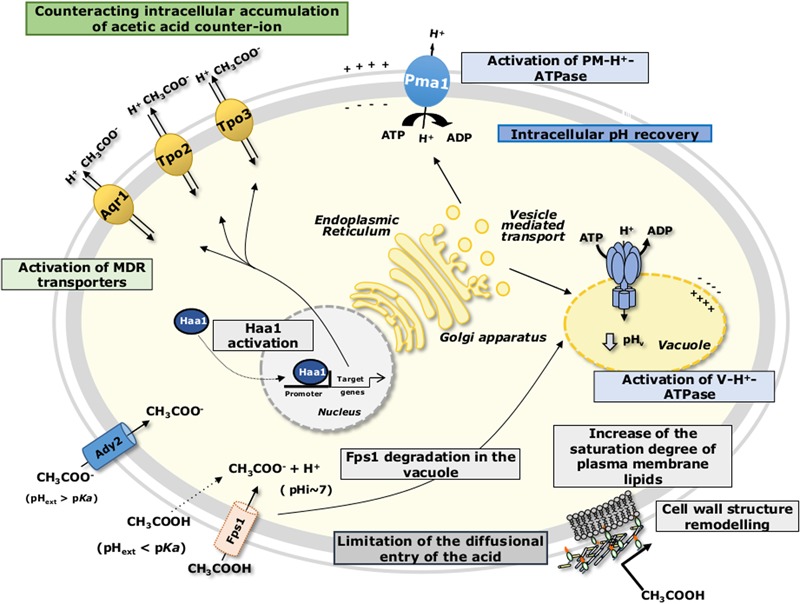
Schematic model for the adaptive response of *S. cerevisiae* to acetic acid-induced stress as detailed in the text. pH_ext_, external pH; pH_i_, intracellular pH.

Acetic acid cellular uptake is dependent both on the extracellular pH and on the specific growth conditions. During *S. cerevisiae* growth in glucose-repressible conditions and at a pH below acetic acid p*K*_a_ (=4.76), acetic acid is mainly in its undissociated form, CH_3_COOH, which is able to passively diffuse across the cell membrane lipid bilayer (Casal et al., 1996). Additionally, the aquaglyceroporin Fps1 was proposed to facilitate the uptake of acetic acid into the yeast cell (Mollapour and Piper, 2007) (**Figure 2**). Once inside the cell, at the near-neutral cytosol, acetic acid dissociates leading to the release of protons (H^+^) and of the negatively charged acetate counterion (CH_3_COO^-^). During growth of derepressed *S. cerevisiae* cells or growth of cells at a pH higher than acetic acid pK_a_, the dissociated form of the acid prevails, and the acid anion is transported at least by the acetate carrier Ady2 (Casal et al., 1996; Paiva et al., 1999, 2004) (**Figure 2**). Once inside the cell, acetate is unable to move back across the plasma membrane by simple diffusion, and accumulates in the cell interior causing increased turgor pressure and severe oxidative stress (Piper et al., 2001). On the other hand, reduction of intracellular pH (pH_i_) caused by the release of protons upon acetic acid dissociation leads to the inhibition of metabolic activity, among other deleterious effects (Pampulha and Loureiro-Dias, 1989, 1990; Orij et al., 2012).

**FIGURE 3:**
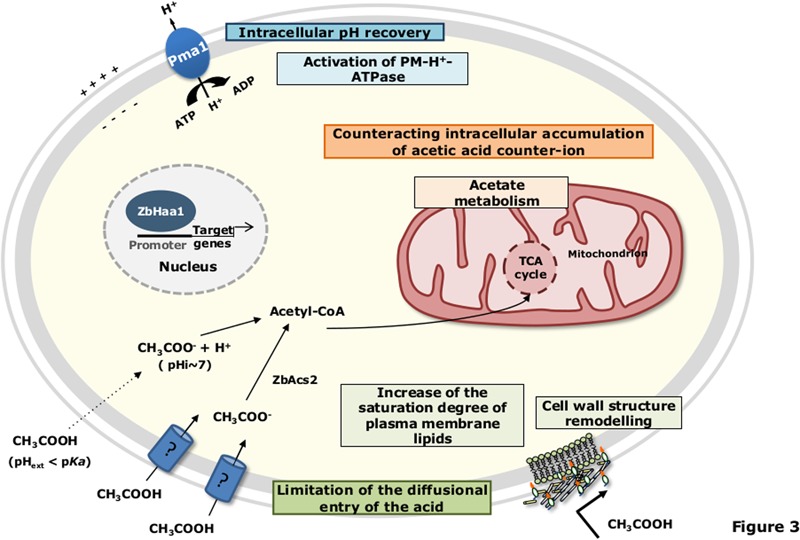
Schematic model for the adaptive response of *Z. bailii* to acetic acid-induced stress. Details are provided in the text. pH_ext_, external pH; pH_i_, intracellular pH; TCA, tricarboxylic acid.

Differently from *S. cerevisiae*, in *Z. bailii* cells cultivated in the presence of both glucose and acetic acid, simple diffusion of the undissociated form of the acid seems to have a minor contribution to the overall uptake of acetic acid (Sousa et al., 1996). Under such experimental conditions or if glucose or fructose are the sole available carbon sources, a non-glucose repressible acetic acid carrier is present and controlled by the intracellular concentration of acetate (Sousa et al., 1996, 1998) (**Figure 3**). When *Z. bailii* cells are cultivated in a medium containing acetic acid as the sole carbon source, acetic acid is transported by a saturable transport system, which is also able to transport propionic and formic acids (Sousa et al., 1996) (**Figure 3**). However, these carriers are not yet identified.

### Intracellular Acidification and pH Recovery

Acidification of the cytosol has been considered one of the major causes of *S. cerevisiae* growth inhibition upon acetic acid stress (Ullah et al., 2012; Stratford et al., 2013a). However, growth inhibition by acetic acid is related not with the initial intracellular acidification levels, but rather with cells’ ability to recover a more physiological pH_i_ (Ullah et al., 2012). In order to counteract the dissipation of the H^+^ gradient across plasma membrane, the yeast cell is strongly dependent on the activation of the plasma membrane H^+^-ATPase (mainly encoded by *PMA1* gene), in coordination with the activation of vacuolar membrane H^+^-ATPase (Carmelo et al., 1997; Ullah et al., 2012; Stratford et al., 2013a) (**Figure 2**). The co-activation of these two proton pumps contributes to the active expulsion of protons accumulated in the cytosol upon acetic acid dissociation, both to the exterior of the cell and to the vacuole lumen (Carmelo et al., 1997; Stratford et al., 2013a) (**Figure 2**). The deletion of genes involved in pH_i_ homeostasis, as for example *VMA1, VMA2, VMA4-8*, and *VMA13* encoding vacuolar ATPase complex proteins confers susceptibility to *S. cerevisiae* cells exposed to sublethal concentrations of acetic acid (Kawahata et al., 2006; Mira et al., 2010b), strongly supporting the idea of the important role of vacuolar ATPase in adaptation to acetic acid. The energy-dependent recovery of pH_i_ in acetic acid-stressed *S. cerevisiae* cells (Pampulha and Loureiro-Dias, 2000; Ullah et al., 2013), together with the inhibition of glycolytic activity induced by the release of protons and subsequent cytoplasmic acidification (Pampulha and Loureiro-Dias, 1990), leads to a reduction in cellular ATP levels. Cells exposed to different levels of acetic acid exhibit different responses in terms of intracellular ATP levels (Ullah et al., 2013). Under more severe acetic acid stress, causing complete growth inhibition, ATP levels were higher than those found in cells subjected to moderate stress, where growth was partially inhibited. This cell strategy of reducing ATP consumption upon severe stress was suggested to be advantageous to maintain energy reserves for later recovery of growth in more favorable conditions (Ullah et al., 2013).

Although the activation of plasma membrane or vacuolar membrane proton ATPases in *Z. bailii* cells exposed to acetic acid stress has never been studied, the activation of plasma membrane H^+^-ATPase activity occurs under benzoic acid stress, leading to the efflux of H^+^ and simultaneous influx and accumulation of K^+^ (Macpherson et al., 2005). These ionic movements were described as well for *S. cerevisiae* in short-term preservative stress (Macpherson et al., 2005).

*Zygosaccharomyces bailii* was suggested to better tolerate short-term decrease of pH_i_ than *S. cerevisiae* (Arneborg et al., 2000), as well as significant pH_i_ drops during exponential phase of growth that are restored afterward in stationary phase (Dang et al., 2012). Apparently, *Z. bailii* employs different strategies to cope with different levels of acetic acid stress (Dang et al., 2012). For more inhibitory acetic acid concentrations, pH_i_ decreases and is maintained around the same value during exponential phase; recovery of pH_i_ to more physiological levels is registered during the stationary phase of growth (Dang et al., 2012). However, when the growth medium is supplemented with a milder inhibitory concentration of acetic acid, there is an initial moderate drop in pH_i_ that is maintained throughout growth, suggesting that cells adapt to the slightly lower pH_i_ to the point where pH_i_ recovery was not required (Dang et al., 2012).

In addition to the responses exhibited by the average yeast cell population, single cell-specific responses to acetic acid have also been investigated in both yeast species. Individual *Z. bailii* cells present in a population exposed to different weak acids exhibit variable tolerance to acetic, sorbic and benzoic acids (Steels et al., 2000; Stratford et al., 2013b). The most tolerant sub-population represents a small fraction of the bulk population and has a lower pH_i_, which leads to reduced intracellular dissociation of any weak acid and, consequently, reduced accumulation of the counterion in the cytoplasm, thus conferring tolerance to any weak acid, but not to other type of inhibitors (Stratford et al., 2013b). This cross-tolerance phenomenon indicates that tolerance is not dependent on the specific acid structure, but rather relies on a mechanism that decreases the uptake and/or accumulation of any weak acid.

Cell-to-cell heterogeneity was also implicated in *S. cerevisiae* tolerance to acetic acid since only the fraction of cells with low initial pH_i_ values was able to recover pH_i_ and resume growth in the presence of the acid (Fernández-Niño et al., 2015). However, the initial pH_i_ is not the only factor influencing *S. cerevisiae* tolerance to acetic acid as shown when two strains with different tolerances to this weak acid were compared (Fernández-Niño et al., 2015).

### Acetate Detoxification Mechanisms

Specific inducible transporters are presumably involved in the active expulsion of acetate from *S. cerevisiae* cell interior. Among them are the plasma membrane transporters of the Major Facilitator Superfamily (MFS), involved in Multidrug/Multixenobiotic Resistance (MDR/MXR) (dos Santos et al., 2014), Tpo2 and Tpo3 (Fernandes et al., 2005; Kawahata et al., 2006; Mira et al., 2010b), and Aqr1 (Tenreiro et al., 2002) (**Figure 2**). Remarkably, *TPO2* was found to be activated in *S. cerevisiae* cells upon acetic acid stress in several genome-wide transcriptional profiling studies (Kawahata et al., 2006; Abbott et al., 2007; Mira et al., 2010a; Bajwa et al., 2013). In contrast to *S. cerevisiae*, no acetate export system has hitherto been suggested or described in *Z. bailii*. Moreover, there are significant differences between *S. cerevisiae* and *Z. bailii* concerning acetate catabolization. In *S. cerevisiae* the use of acetate as a carbon source is in general repressed by glucose, and cells cultivated in a medium containing both glucose and acetic acid exhibit diauxic growth with acetic acid only being consumed after glucose has been exhausted from the medium (Casal et al., 1996, 1998; Vilela-Moura et al., 2011). When acetic acid is the sole carbon source present in the medium, this weak acid is consumed through its conversion to acetyl coenzyme A (acetyl-CoA), catalyzed by two acetyl-CoA synthetases, Acs1 (peroxisomal) or Acs2 (cytosolic) (dos Santos et al., 2003). The acetyl-CoA produced from acetate then enters the mitochondria to be oxidized in the tricarboxylic acid (TCA) cycle or remains outside the mitochondria to be metabolized in the glyoxylate cycle, which replenishes the cell with succinate, a crucial metabolite to produce different biosynthetic precursors (dos Santos et al., 2003). *Z. bailii* has the ability to catabolize acetate even in the presence of glucose (Sousa et al., 1998; Guerreiro et al., 2012; Rodrigues et al., 2012). This mechanism involves the regulation of both acetic acid membrane transport and acetyl-CoA synthetase activity, allowing the maintenance of an intracellular concentration of the acetate below toxic levels (Rodrigues et al., 2004; Sousa et al., 1996). Other proteins related to carbohydrate metabolism (Mdh1, Aco1, Cit1, Idh2 and Lpd1) and energy generation (Atp1 and Atp2), as well as general and oxidative stress response (Sod2, Dak2, Omp2), are also involved in acetate catabolization in *Z. bailii* (Guerreiro et al., 2012). This strengthens the concept that glucose and acetic acid are co-consumed in *Z. bailii*, with acetate being channeled into the TCA. This behavior was hypothesized to contribute to the remarkable tolerance of *Z. bailii* to acetic acid, particularly in environments that are rich in both carbon sources, such as during vinification (Sousa et al., 1998) or fermentation of lignocellulosic hydrolysates (Jönsson et al., 2013).

### Acetic Acid-Induced Alterations of the Cellular Envelope

It has been proposed that remodeling of cell wall and/or plasma membrane structure may occur and represent one of the most important adaptive mechanisms of tolerance to weak acids (**Figures 2, 3**) (Simões et al., 2006; Mollapour et al., 2009; Lindberg et al., 2013; Guerreiro et al., 2016a). Indeed, reducing cellular envelope permeability by altering cell wall and plasma membrane chemical structure and properties, thereby decreasing weak acid diffusion, is a much more energetically efficient method than relying on the active extrusion of protons and acid. Consistent with this concept, multiple genes involved in the synthesis of cell wall polysaccharides, cell wall structure assembly and remodeling, and in sphingolipid and sterol biosynthetic pathways were demonstrated to be determinants of tolerance, or to be transcriptionally responsive to acetic acid in *S. cerevisiae*, in several genome-wide studies (Kawahata et al., 2006; Abbott et al., 2007; Mira et al., 2010a,b; Lee et al., 2015; Longo et al., 2015).

Among the proteins found to mediate the alteration of cell wall structure in response to weak acids in *S. cerevisiae*, is Spi1, a glycosylphosphatidylinositol-anchored cell wall protein, which is particularly important for tolerance to lipophilic acids like benzoic or octanoic (Simões et al., 2006). Although the expression of *SPI1* was considered not significant in the adaptation and tolerance to acetic acid (Simões et al., 2006), *SPI1* transcription was activated in response to this acid in several transcriptional analyses (Kawahata et al., 2006; Abbott et al., 2007; Mira et al., 2010a), thereby suggesting Spi1 as an important player in response to acetic acid. Although the specific function of *YGP1*, encoding a cell wall-related secretory glycoprotein, is still unknown, this gene is also considered a key player in *S. cerevisiae* tolerance to acetic acid, since it is activated in cells exposed to acetic acid (Kawahata et al., 2006; Abbott et al., 2007; Mira et al., 2010a) and the mutant with this gene deleted is very susceptible to this acid (Mira et al., 2010b). *Z. bailii YGP1* homologue was also found to be up-regulated in response to acetic acid (Palma et al., 2017a).

**FIGURE 4:**
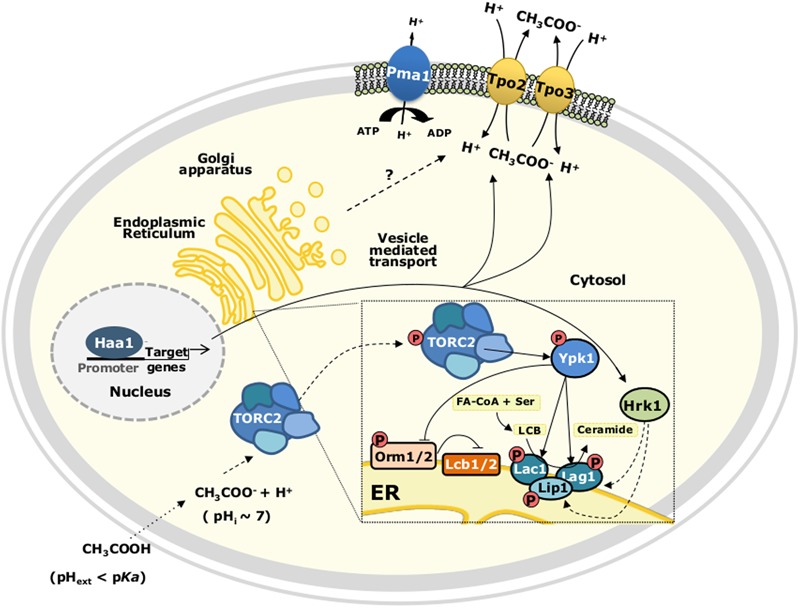
Proposed model for TORC2-Ypk1 dependent activation of the sphingolipid biosynthetic pathway in acetic acid-stressed *S. cerevisiae* cells. TORC2, Target of Rapamycin (TOR) Complex 2; FA-CoA, fatty acyl-CoA; LCB, long chain base.

Major alterations occurring in the plasma membrane lipid composition are also involved in yeast tolerance to acetic acid. A lipidomic analysis revealed that, for both *S. cerevisiae* and *Z. bailii*, during aerobic growth in bioreactors, the supplementation of the growth medium with acetic acid induced significant changes in the cellular lipid content (Lindberg et al., 2013). In acetic acid-stressed *Z. bailii* cells, the total amount of glycerophospholipids (GPLs) was found to be slightly lower than in *S. cerevisiae*, while the degree of saturation of GPLs was increased under these same conditions (Lindberg et al., 2013). Increased levels of complex sphingolipids were detected for both species in the mid-exponential phase of acetic acid-adapted growth (Lindberg et al., 2013). Remarkably, the basal level of complex sphingolipids was significantly higher in *Z. bailii* than in *S. cerevisiae* leading the authors to suggest a link between high sphingolipid levels and the intrinsic tolerance of *Z. bailii* species to acetic acid (Lindberg et al., 2013). The correlation between the fraction of sphingolipids and membrane permeability to acetic acid was further investigated and confirmed based on *in silico* simulations of model membranes (Lindahl et al., 2016). Sphingolipids are essential structural components of cellular membranes, in particular the plasma membrane, playing important roles in signaling and intracellular trafficking, as well as in the regulation of diverse processes (Dickson, 2008). The importance of the regulation of sphingolipid biosynthetic pathway in *S. cerevisiae* response and tolerance to acetic acid was more recently examined during *S. cerevisiae* early adaptive response to acetic acid (Guerreiro et al., 2016a). It was demonstrated that Ypk1 phosphorylation and activation by the membrane-localized protein kinase complex TORC2 is stimulated in response to acetic acid stress, consequently activating lipid synthesis (Guerreiro et al., 2016a) (**Figure 4**). Several plasma membrane lipid and protein homeostasis processes are regulated by the protein kinase Ypk1 (reviewed in Roelants et al., 2017). For instance, TORC2/Ypk1 signaling was proposed to inactivate by phosphorylation the endoplasmic reticulum-associated Orm1/2 protein inhibitors of L-serine:palmitoyl-CoA acyltransferase enzyme complex Lcb1/2 that catalyzes the first step of sphingolipid biosynthesis in response to compromised sphingolipid synthesis, thus restoring sphingolipid biosynthesis (Roelants et al., 2011). In *S. cerevisiae* cells exposed to acetic acid Ypk1 phosphorylates Orm1 and two functionally redundant isoforms of the ceramide synthase complex, Lac1 and Lag1 (Guerreiro et al., 2016a) (**Figure 4**).

In what concerns ergosterol content in acetic acid-challenged cells, the levels of this sterol decreased in *S. cerevisiae*, but not in the more tolerant species *Z. bailii* (Lindberg et al., 2013). Ergosterol is the major sterol present in the plasma membrane and has been shown to have vital functions in *S. cerevisiae* cells, affecting its membrane fluidity and permeability (Abe and Hiraki, 2009). In fact, the level of ergosterol present in the plasma membrane plays a crucial role in *S. cerevisiae* tolerance to several stresses (Swan and Watson, 1998; Higgins et al., 2003; Dupont et al., 2011; Henderson and Block, 2014), and multiple genes involved in sterols biosynthetic pathways were demonstrated to be determinants of tolerance or to be transcriptionally responsive to acetic acid (Mira et al., 2010a,b).

## Transcriptional Regulatory Networks Controlling Adaptive Response and Tolerance to Acetic Acid

### Genome-Wide Transcriptional Regulation Induced by Acetic Acid Stress in *S. cerevisiae*

Yeast response to stressful conditions relies on the activation of general or specific regulatory pathways that determine cell fate, either to adapt and survive in the hostile environment or to die. *S. cerevisiae* cells respond to several different external insults by altering the transcription level of a particular set of genes of the generally called Environmental Stress Response (ESR), mainly controlled by Msn2 and Msn4 transcriptional activators (Gasch et al., 2000). The activation of genes from the ESR program was also registered when *S. cerevisiae* cells are exposed to several stresses relevant in Food Industry and Industrial Biotechnology, in particular in the response to acetic acid (Kawahata et al., 2006; Abbott et al., 2007; Li and Yuan, 2010; Mira et al., 2010a) and/or other weak acids (Schüller et al., 2004; Abbott et al., 2007; Mira et al., 2009). In addition to the general stress response transcription factors Msn2/Msn4, other regulators are involved in the genome-wide transcriptional response of *S. cerevisiae* to weak acid-induced stress (reviewed in Mira et al., 2010c; Teixeira et al., 2011). Specifically, War1, responsible for the induction of *PDR12* transcription and therefore being crucial for response and tolerance to propionic, benzoic and sorbic acids (Piper et al., 2001; Schüller et al., 2004), Rim101, required for maximal tolerance to weak acid-induced stress, including acetic acid (Mira et al., 2009), and Haa1, required for adaptation and tolerance to the more hydrophilic formic, acetic, lactic and propionic acids (Fernandes et al., 2005; Abbott et al., 2007; Henriques et al., 2017). In yeast cells exposed to stresses that lead to mitochondrial dysfunction, it is the mitochondrial retrograde (RTG) signaling pathway that establishes mitochondria-to-nucleus communication, regulating the necessary alteration of nuclear gene expression (Butow and Avadhani, 2004), which is mainly controlled by the transcriptional regulators Rtg1 and Rtg3 (Sekito et al., 2000). The integration of genome-wide data from transcriptomic profiling of *S. cerevisiae* response and tolerance to several weak acids has contributed to the understanding of cellular responses to weak acid-induced stress as a dynamic system, where each transcription factor-associated network can cross-talk with others (Mira et al., 2010c). Since Haa1 is considered the primary regulator of *S. cerevisiae* transcriptional response to acetic acid (Mira et al., 2010a), the next two sections are dedicated to this transcription factor and to its *Z. bailii* ortholog ZbHaa1.

### The Haa1 Regulon as the Main Player in the Control of *S. cerevisiae* Response to Acetic Acid

Haa1 was first identified based on the homology and structural similarity with the DNA binding domain (DBD) of the copper-regulated transcription factor Cup2 (alias Ace1) (Hu et al., 1990; Keller et al., 2001). The paralog pair Haa1 and Cup2 DBDs comprise 123 and 124 amino acid residues, respectively, at the N-terminal and include a conserved zinc module and a set of four cysteine-cysteine clusters organized in a consensus sequence that forms the copper regulatory domain (CuRD). Such conservation at the level of the DBD led to the hypothesis that, like Cup2, Haa1 could play a role in copper homeostasis (Keller et al., 2001). However, metalloregulation and the involvement of Haa1 in S. *cerevisiae* tolerance to copper could not be associated to this transcription factor (Keller et al., 2001). Later, it was attributed for the first time a function to Haa1 as having an essential role in *S. cerevisiae* adaptation and tolerance to weak acids, especially to short-chain hydrophilic acids such as acetic acid (Fernandes et al., 2005). The alterations detected in yeast genomic expression during early response to acetic and lactic acids highlighted the involvement of Haa1 in the transcriptional reprogramming of *S. cerevisiae* cells during the adaptive response to these weak acids (Abbott et al., 2007; Mira et al., 2010a). Haa1 is required for the transcriptional activation of approximately 80% of the acetic acid-responsive genes, and thus proposed as being the main player in yeast genomic expression regulation under acetic acid stress (Mira et al., 2010a). Following Haa1-responsive element (HRE) identification (Mira et al., 2011), HRE was found to be present in the promoter region of about 55% of the genes whose expression is activated in response to acetic acid under the dependence of Haa1, suggesting that these genes are direct targets of this transcription factor (Mira et al., 2010a). The remaining Haa1-dependent acetic acid responsive genes are presumably indirectly regulated by Haa1 (Mira et al., 2010a, 2011), for example through the action of other genes encoding transcription factors directly regulated by Haa1 (Mira et al., 2010a, 2011). This is the case of Msn4, mediating the general stress response in yeast (Gasch et al., 2000), Fkh2, implicated in yeast response to oxidative stress (Postnikoff et al., 2012), and the transcriptional repressor Nrg1, also involved in yeast response to several stresses (Vyas et al., 2005; Mira et al., 2010a, 2011). These regulatory associations dependent on Haa1 upon weak acid-induced stress were highlighted recently in the upgrade of YEASTRACT database (Teixeira et al., 2017).

**FIGURE 5:**
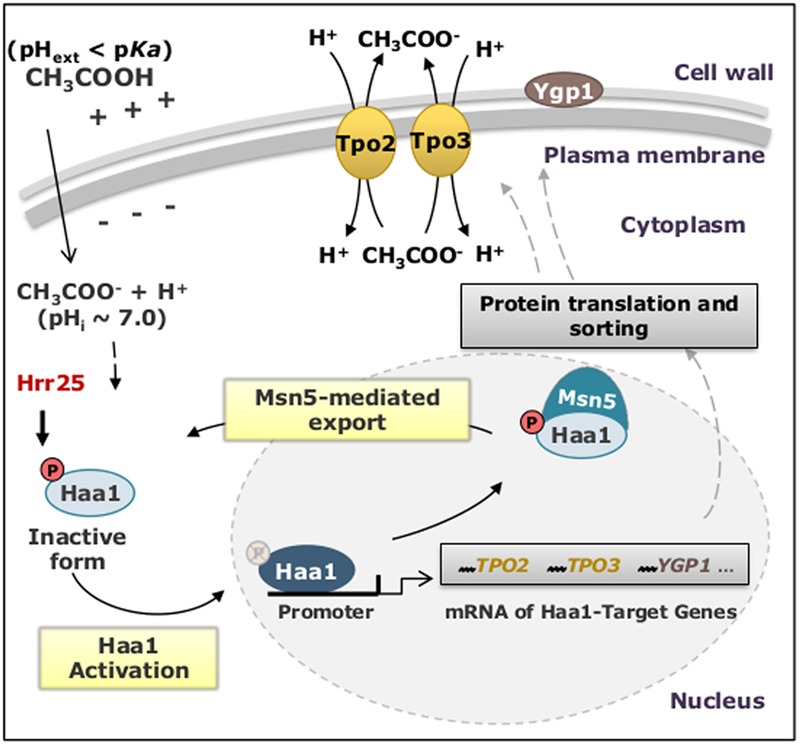
Schematic model of the mechanisms proposed to regulate Haa1 activity in *S. cerevisiae*, in response to lactic and acetic acid-induced stress. The description of the proposed events is detailed in the text. pH_ext_, external pH; pH_i_, intracellular pH.

The biological activity of Haa1 was found to be regulated by its sub-cellular localization that, in turn, is regulated by Haa1 phosphorylation levels (Sugiyama et al., 2014). The rapid translocation of Haa1 from the cytosol to the nucleus, where it activates the transcription of its target-genes in response to lactic (Sugiyama et al., 2014) or acetic (Swinnen et al., 2017) acids, is concomitant with a decrease in Haa1 phosphorylation levels (Sugiyama et al., 2014). The casein kinase I isoform Hrr25 is an important negative regulator of Haa1, inhibiting this transcription factor’s activity by phosphorylation (Collins et al., 2017). It was also demonstrated that the exportin Msn5, which preferentially exports phosphorylated cargo proteins, interacts with Haa1 being essential to its exit from the nucleus where its function as transcription factor takes place (Sugiyama et al., 2014) (**Figure 5**).

Among the genes of the Haa1-regulon whose expression was found to confer yeast protection against acetic acid are protein kinases, proteins involved in lipid metabolism (sphingolipids most notably ceramides, which are bioactive signaling molecules known to play a crucial role in lipid-based signaling in yeast response to stress), in nucleic acid processing, multidrug resistance transporters and proteins of unknown function. The expression of *SAP30* (encoding a subunit of the Rpd3L histone deacetylase complex) and *HRK1* provided the strongest protective effect toward acetic acid (Mira et al., 2010a). Even though the first biological function attributed to Hrk1 was the activation of the plasma membrane proton pump Pma1 in response to glucose metabolism (Goossens et al., 2000), its mild effect on Pma1 activity suggests that this kinase could play other roles in yeast. The fact that *hrk1*Δ cells showed a marked increase in the intracellular concentration of radiolabeled acetic acid and that Hrk1 belongs to a family of kinases dedicated to the regulation of plasma membrane transporters, led to hypothesize that Hrk1 could be involved in the regulation of one or more plasma membrane acetate exporters, such as the MDR/MXR transporters Tpo3, Tpo2 and Aqr1, all known determinants of tolerance to this weak acid (Mira et al., 2010a,b). To elucidate the biological role of Hrk1, the effect of Hrk1 expression in yeast membrane associated-phosphoproteome was examined in *S. cerevisiae* parental and *hrk1*Δ cells exposed, or not, to sublethal concentrations of acetic acid (Guerreiro et al., 2017). In this study, the MDR/MXR transporters Tpo3 and Tpo4 were found to exhibit altered phosphorylation in response to acetic acid stress under the dependence of Hrk1. This evidence led to hypothesize that the Hrk1-mediated phosphorylation of Tpo3 may contribute to regulate the activity of this drug pump (Guerreiro et al., 2017), thereby lowering the accumulation of acetic acid in the parental strain when compared to *hrk1*Δ or *tpo3*Δ cells, as previously described (Fernandes et al., 2005; Mira et al., 2010a). Other important membrane proteins in the context of response and tolerance to acetic acid-induced stress are present in the published dataset and certainly deserve further studies (Guerreiro et al., 2017).

### The *Z. bailii* Haa1 (ZbHaa1) Is Required for Acetic Acid and Copper Stress Responses

The importance of ZbHaa1 in *Z. bailii* response and tolerance to acetic acid was demonstrated using the strain *Z. bailii* IST302 that proved to be susceptible to genetic engineering and has the genome fully sequenced (Palma et al., 2015, 2017a). ZbHaa1 was found to be a functional homolog of Haa1 by rescuing the acetic acid susceptibility phenotype of *S. cerevisiae haa1*Δ. Moreover, the disruption of *ZbHAA1* and the expression of an extra *ZbHAA1* copy in *Z. bailii* confirmed *ZbHAA1* as a determinant of acetic acid tolerance in this yeast species. The expression of *ZbHAA1* was found to be required for acetic acid stress-induced transcriptional activation of *Z. bailii* genes homologs to the demonstrated *S. cerevisiae* Haa1-target genes: *HRK1, TPO3, MSN4, YGP1, YRO2* and *HSP30* (Palma et al., 2017a). Remarkably, ZbHaa1 (the single ortholog of *S. cerevisiae* Haa1 and Cup2) was demonstrated to have a role in metalloregulation, being involved in copper tolerance and copper-induced transcriptional regulation, a role associated to *S. cerevisiae* Cup2, but not to Haa1 (Palma et al., 2017a). Phylogenetic and gene neighborhood analyses suggested the subfunctionalization of *Z. bailii* ancestral bifunctional protein Haa1/Cup2 after the whole-genome duplication event, originating *S. cerevisiae* Haa1 and Cup2 paralogs. As found for *S. cerevisiae*, ZbHaa1 is likely a candidate molecular target for the design of new strategies to overcome *Z. bailii* spoilage in foods and beverages.

## Mechanisms Involved in Yeast Response to Lethal Concentrations of Acetic Acid

High concentrations of acetic acid are able to induce in *Z. bailii* either an apoptotic or a necrotic death process, depending on the acid concentration present in the medium, as observed for *S. cerevisiae* (Ludovico et al., 2001, 2003). However, since *Z. bailii* is highly resistant to acetic acid-induced cell death, this effect is observed at much higher concentrations of the acid for this yeast species (range of 320–800 mM, pH 3.0) than in *S. cerevisiae* (20–120 mM, pH 3.0) (Ludovico et al., 2001, 2002, 2003). The main known mechanisms involved in acetic acid-induced RCD were also much more extensively studied in *S. cerevisiae* than in *Z. bailii*.

In *S. cerevisiae*, acetic acid is known to induce a RCD process with an apoptotic phenotype that is dependent on mitochondria. Indeed, apart from its bioenergetic function, mitochondria have an essential role in the decision of cells’ life or death (reviewed in Ždralević et al., 2012) and many of the hallmarks involved in yeast mitochondria-dependent RCD process with an apoptotic phenotype have been characterized, including the translocation of pro-apoptotic factors (e.g., cytochrome *c*) from the mitochondria to the cytosol, phosphatidylserine externalization to the outer layer of the cytoplasmic membrane, production and consequent build-up of mitochondrial reactive oxygen species (ROS), DNA fragmentation and chromatin condensation (reviewed in Carmona-Gutierrez et al., 2010; Guaragnella et al., 2012).

Concerning tolerance to acetic acid-induced RCD, the activation of the RTG signaling pathway was proposed to be involved when *S. cerevisiae* cells were cultivated in a non-repressible carbon source such as raffinose (Guaragnella et al., 2013) or previously adapted to low pH environments (Giannattasio et al., 2005). In yeast, the mitochondrial RTG pathway acts in parallel with the TOR and the Ras-cAMP pathways in the regulation of acetic acid-induced RCD (Phillips et al., 2006; Almeida et al., 2009; Giannattasio et al., 2013).

In *Z. bailii* the morphological changes observed during RCD induced by high concentrations of acetic acid show extensive mitochondrial ultrastructural changes during the RCD process that were not seen for *S. cerevisiae* when equivalent deleterious concentrations were used (Ludovico et al., 2003). The acetic acid-induced RCD process with an apoptotic phenotype was also characterized in *Z. bailii* by the maintenance of plasma membrane integrity, DNA fragmentation, ROS production, and cytochrome *c* translocation from the mitochondria into the cytosol (Ludovico et al., 2003; Guerreiro et al., 2016b). The global mitochondrial proteomic response to acetic acid-induced RCD in *Z. bailii* hybrid strain ISA1307 was examined by 2-DE quantitative proteomics (Guerreiro et al., 2016b). The increase of different ROS (namely H_2_O_2_ and superoxide anion) observed in *Z. bailii* cells undergoing RCD induced by acetic acid, coupled with different changes in abundance of several antioxidant enzymes observed in that same cell population, suggest that dynamic modulation of ROS might be taking place in cells exposed to acetic acid concentrations that induce RCD (Guerreiro et al., 2016b). Nevertheless, the effectors that play a role in acetic acid-induced RCD remain poorly characterized.

## Physiological Genomics-Guided Strategies to Improve Acetic Acid Tolerance

The topics covered in this review show that acetic acid tolerance phenotype is complex and multifactorial since it requires coordinated changes at several levels in the cell. For this reason, some of the most promising strategies being used to obtain more robust industrial strains involve the manipulation of genes that play a crucial role in the regulatory cascades controlling stress tolerance and the exploitation of genome engineering strategies, which allows the generation of diversity and subsequent selection of the strains that possess the trait of interest. The exploitation of this and other strategies are reviewed in the next sections.

### Overexpression or Deletion of Single Genes

The genetic engineering of laboratory *S. cerevisiae* strains through the overexpression of single genes has yielded strains with increased acetic acid tolerance. Specifically, the overexpression of *WHI2*, encoding a protein required for full activation of the general stress response (Chen et al., 2016b), of the genes *PEP3*, encoding a vacuolar membrane protein involved in vesicular tethering/docking/fusion, *STM1*, encoding a protein required for optimal translation under nutrient stress, *PEP5*, encoding the E3 ubiquitin protein-ligase involved in the catabolism of histones (Ding et al., 2015a), or the gene *ACS2*, encoding acetyl-coA synthetase isoform (Ding et al., 2015b), decreased the duration of lag phase of *S. cerevisiae* cells cultured with acetic acid. Improvement of *S. cerevisiae* growth and alcoholic fermentation performance in the presence of acetic acid also resulted from the overexpression of *SET5* and *PPR1*, coding for a methyltransferase for the methylation of histone H4 at Lys5, -8, and -12 and a transcription factor involved in the regulation of pyrimidine pathway, respectively (Zhang et al., 2015). The overexpression of *ASC1* (G-protein beta subunit), *GND1* (6-phosphogluconate dehydrogenase) (Lee et al., 2015), *PMA1* (Lee et al., 2017) or *COX20* (cytochrome oxidase chaperone) (Kumar et al., 2015) also increased *S. cerevisiae* tolerance to acetic acid.

*Saccharomyces cerevisiae* increased robustness to acetic acid has also been accomplished through expression of *Z. bailii* genes *ZbMSN4, ZbTIF3* (Palma et al., 2015), encoding the homologs of *S. cerevisiae* general stress response transcription factor and translation initiation factor, respectively, or *ZbHAA1* (Palma et al., 2017a). The overexpression of these genes in *Z. bailii* were also found to increase its tolerance to acetic acid (Palma et al., 2015).

An increase in *S. cerevisiae* tolerance to acetic acid was also demonstrated by individual deletion of several genes. Specifically, the deletion of *HSP82* (protein chaperone), *ATO2* (putative transmembrane protein involved in export of ammonia) and *SSA3* (ATPase involved in protein folding and the response to stress) increased *S. cerevisiae* tolerance to acetic acid, presumably by contributing indirectly to enhanced proton export and diminished levels of H_2_O_2_ within the cell upon acetic acid stress (Lee et al., 2015). The deletion of *RTT109* (histone acetyltransferase) also increased *S. cerevisiae* tolerance to acetic acid, which was suggested to be related to the activation of transcription of stress responsive genes and to increased resistance to oxidative stress (Cheng et al., 2016). In addition, the elimination of *JJJ1* (co-chaperone) enhanced acetic acid tolerance, which was proposed to be related to increased levels of long-chain fatty acids and trehalose in the cell, together with an increase in catalase activity (Wu et al., 2016). Remarkably, a single mutation in cytochrome *c* (the substitution of the highly conserved residue tryptophan 65 by a serine) that impaired electron transfer to the functional cyt *c* oxidase, was found to lead to increased cellular viability upon acetic acid stress (Guaragnella et al., 2011).

### Manipulation of Haa1-Regulon in *S. cerevisiae*

The manipulation of the Haa1-regulon, namely through *HAA1* overexpression (Tanaka et al., 2012; Inaba et al., 2013; Sakihama et al., 2015; Chen et al., 2016a) or *HAA1* mutation (Zahn and Jacobson, 2015; Swinnen et al., 2017) was shown to increase tolerance to acetic acid. In a first study, the *HAA1* gene was placed under the control of the constitutive *TDH3* promoter (HAA1-OP strain) (Tanaka et al., 2012). This promoter exchange led to increased *HAA1* transcript levels, as well as of four of the Haa1-regulated genes (*TPO2, TPO3, YRO2* and *YGP1*) even when cells were grown in the absence of acetic acid stress (Tanaka et al., 2012). The resulting strain, HAA1-OP, showed increased tolerance to acetic acid and decreased intracellular acetic acid accumulation when compared with the parental strain (Tanaka et al., 2012). A similar approach was used in an attempt to improve the industrial strain Ethanol-Red (ER). The resulting diploid ER HAA1-OP strain (with *HAA1* transcription being under the control of the *TDH3* promoter) showed increased *HAA1* transcription levels in non-stress conditions, and was also less susceptible to acetic acid-induced stress than the corresponding parental strain (Inaba et al., 2013). *HAA1* was also overexpressed from a multicopy plasmid in a successful attempt to increase ethanol productivity during xylose fermentation in the presence of acetic acid (Sakihama et al., 2015). Likewise, *HAA1* overexpression improved specific sugar consumption and cell growth rate in the presence of acetic acid when compared with the non-transformed strain (Chen et al., 2016a). Based on error-prone PCR of the *HAA1* coding sequence, a highly tolerant mutant allele holding two point mutations was identified as being able to improve the acetic acid tolerance of *S. cerevisiae* (Swinnen et al., 2017). Following dissection of the individual contribution of each mutation, it was found that the major improvement in acetic acid tolerance was caused by a single amino acid exchange at position 135 (serine to phenylalanine). Remarkably, the transcriptional levels of four Haa1-target genes, selected at random, significantly increased in acetic acid-challenged cells of the strain harboring the single Haa1 mutation when compared to cells of the parental strain (Swinnen et al., 2017). In a patented work, mutagenesis of the Haa1 at the level of its transactivation domain was also found to increase the activity to this transcription factor, with a consequent increase in the yield of ethanol produced during fermentation of lignocellulose hydrolysates containing acetic acid (Zahn and Jacobson, 2015). All these studies suggest that the genetic manipulation of the Haa1 pathway is suitable to obtain more robust *S. cerevisiae* strains in an industrial context.

### *S. cerevisiae* Evolutionary Engineering and Genome Shuffling

The use of evolutionary engineering strategies, usually focusing on selection of a unique genetic trait responsible for that advantageous phenotype (Wright et al., 2011; Koppram et al., 2012), were also successfully employed for the improvement of *S. cerevisiae* tolerance to acetic acid. For example, a novel laboratory evolution strategy based on alternating cultivation cycles in the presence or absence of acetic acid was recently described as conferring a selective advantage to cells that are constitutively tolerant to acetic acid (González-Ramos et al., 2016). Mutations in four genes (*ASG1, ADH3, SKS1* and *GIS4*) were identified in this study as being implicated in the constitutive acetic acid tolerance phenotype of the evolved strains (González-Ramos et al., 2016). More recently, an evolutionary engineering study involving the cross of a strain with high acetic acid tolerance with an industrial reference strain, followed by multiple rounds of inbreeding of the resulting haploid segregants and quantitative trait loci (QTL) mapping, envisaged the study of the polygenic nature of the high acetic acid tolerance phenotype (Meijnen et al., 2016). This study allowed the identification of a mutated *HAA1* allele (serine to asparagine amino acid substitution at position 506) responsible for the superior character of the segregant strain under acetic acid stress (Meijnen et al., 2016). Among the novel genes identified in this study as contributing to high acetic acid tolerance is *DOT5*, encoding a nuclear thiol peroxidase and functioning as an alkyl-hydroperoxide reductase agent during post-diauxic growth. Remarkably, this gene also proved to play an important role in acetic acid tolerance in a study that also mapped the QTLs of segregants obtained from inbreeding of two industrial strains with distinct acetic acid tolerance phenotypes (Geng et al., 2016). Genome shuffling has also emerged as an alternative experiment strategy to improve tolerance to several stressors, including acetic acid (Zheng et al., 2011).

### *S. cerevisiae* Transcriptome Remodeling

The remodeling of the yeast transcriptome through global transcription machinery engineering has also been applied successfully to obtain acetic acid-tolerant strains. This was achieved by re-programming the cell transcriptome through mutations in *SPT15*, encoding the TATA-binding protein, followed by screening of the mutants with improved tolerance to acetic acid (An et al., 2015). Another example involved the transformation of a yeast strain with an artificial zinc finger protein transcription factor library and subsequent selection of acetic acid-tolerant strains (Ma et al., 2015). Remodeling of transcription through introduction of point mutation in H3/H4 histones (Liu et al., 2014), as well as the generation of extensive alterations in mRNA metabolism through mutagenesis of the poly(A) binding protein encoding gene, *PAB1* (Martani et al., 2015), have also led to the development of strains with improved robustness against acetic acid stress.

### Supplementation of Growth Media with Cations

*Saccharomyces cerevisiae* tolerance to acetic acid can be alleviated by changing the composition of the growth media. The uptake of ions is essential in acetic acid tolerance, and several genes involved in ion homeostasis, for instance potassium transporters, were identified as determinants of tolerance to this weak acid (Kawahata et al., 2006; Mira et al., 2010b). This evidence led the authors to suggest and confirm that potassium supplementation of the growth medium may decrease acetic acid-induced growth inhibition (Mira et al., 2010b). Indeed, potassium is essential for many physiological functions, such as regulation of pH_i_, maintenance of plasma membrane potential, protein synthesis, and enzyme activation (Ariño et al., 2010), which are biological processes highlighted in this review as playing a role in response and tolerance to acetic acid. The increase of extracellular concentration of potassium was also found to be beneficial to increase the tolerance to higher alcohols and ethanol production in both commercial and laboratory strains (Lam et al., 2014). The supplementation of the growth medium with zinc sulfate also improved *S. cerevisiae* tolerance to acetic acid, with zinc presumably acting as an anti-oxidative agent (Wan et al., 2015). The addition of zinc and other metal ions (Mg^2+^ and Ca^2+^) was also associated to an increase of *S. cerevisiae* tolerance toward acetic acid (Ismail et al., 2014). The comparison of the transcriptional profiling of cultures supplemented or not with each of the three metal ions and acetic acid suggested that these ions are involved in the regulation of different genes, and that the up-regulation of cell wall and membrane genes is related with the increased tolerance to acetic acid upon metal ion supplementation (Ismail et al., 2014).

## Concluding Remarks and Future Perspectives

The mechanisms underlying the adaptive response and tolerance to acetic acid in *S. cerevisiae* and *Z. bailii* have been enlightened over the past two decades based on functional and comparative genomics strategies. The knowledge gathered in the mechanisms of yeast response and tolerance to acetic acid have been mostly explored in the development of acetic acid tolerant industrial strains, rather than the in design of novel food preservation technologies. Future manipulation of *S. cerevisiae* tolerance to acetic acid will continue to rely on increasing industrial strains robustness, either through the genetic manipulation of genes that play a crucial role in the regulatory cascades that control stress tolerance, or through genome-scale engineering, thereby allowing the generation of strain diversity and subsequent selection of the strains that possess the trait of interest. Nevertheless, caution is needed during the application of the abovementioned strategies, considering that the impact of the modifications introduced in the engineered strains must be carefully evaluated regarding the potential changes they might cause in important industrial properties of that strain (Deparis et al., 2017). Moreover, many of the successful improvement studies described so far have used low acetic acid tolerant laboratory strains, but their usefulness when applied to highly acetic acid tolerant industrial strains remains to be proved.

Understanding the mechanisms of tolerance to acetic acid in *S. cerevisiae* and *Z. bailii* has undoubtedly brought to light the interspecies diversity and complexity of those mechanisms, which rely on several molecular and physiological responses orchestrated by the expression of multiple genes. Although the model yeast *S. cerevisiae* has been at the forefront of numerous molecular, physiological and genome-wide studies on acetic acid response and tolerance, the release of *Z. bailii* genome annotated sequences and the availability of new *Z. bailii* strains more prone to genetic and laboratory manipulations and of new molecular genetic tools, such as the genome editing tool CRISPR/Cas9, is changing this paradigm. Extensive transcriptomic profiling studies are expected to emerge to characterize the transcriptional regulatory networks underlying *Z. bailii* response and adaptation to acetic acid stress. Given the described relevance of ZbHaa1, the identification and manipulation of the ZbHaa1-signaling pathway in acetic acid challenged *Z. bailii* cells is anticipated as a promising strategy to identify novel molecular targets in this food spoilage yeast species that can also be regarded as potential cell factory for the overproduction of organic acids.

The integrative perspective of the cellular processes described herein has benefited from the exploitation of a systems microbiology approach. They are granting the rational design of strategies to improve alcoholic fermentation processes when yeasts are used as microbial factories.

## Author Contributions

All authors listed have made a substantial, direct and intellectual contribution to the work, and approved it for publication.

## Conflict of Interest Statement

The authors declare that the research was conducted in the absence of any commercial or financial relationships that could be construed as a potential conflict of interest.
